# Trends in Severe Maternal Morbidity in the US Across the Transition to *ICD-10-CM/PCS* From 2012-2019

**DOI:** 10.1001/jamanetworkopen.2022.22966

**Published:** 2022-07-28

**Authors:** Ashley H. Hirai, Pamela L. Owens, Lawrence D. Reid, Catherine J. Vladutiu, Elliott K. Main

**Affiliations:** 1 Maternal and Child Health Bureau, Health Resources and Services Administration, US Department of Health and Human Services, Rockville, Maryland; 2 Center for Financing, Access and Cost Trends, Agency for Healthcare Research and Quality, US Department of Health and Human Services, Rockville, Maryland; 3 California Maternal Quality Care Collaborative, Stanford University, Palo Alto, California

## Abstract

**Importance:**

Surveillance of severe maternal morbidity (SMM) is critical for monitoring maternal health and evaluating clinical quality improvement efforts.

**Objective:**

To evaluate national and state trends in SMM rates from 2012 to 2019 and potential disruptions associated with the transition to *International Classification of Diseases, 10th Revision, Clinical Modification and Procedure Coding System* (*ICD-10-CM/PCS*) in October 2015.

**Design, Setting, and Participants:**

This repeated cross-sectional analysis examined delivery hospitalizations from 2012 through 2019 in the Healthcare Cost and Utilization Project’s National Inpatient Sample and State Inpatient Databases, an all-payer compendium of hospital discharge records from community, nonrehabilitation hospitals. Trends were evaluated using segmented linear binomial regression models that allowed for discontinuities across the *ICD-10-CM/PCS* transition. Analyses were completed from April 2021 through March 2022.

**Exposures:**

Time, *ICD-10-CM/PCS* coding system, and state.

**Main Outcomes and Measures:**

SMM rates, excluding blood transfusion, per 10 000 delivery hospitalizations, overall and by indicator.

**Results:**

From 2012 to 2019, there were 5 964 315 delivery hospitalizations in the national sample representing a weighted total of 29.8 million deliveries with a mean (SD) maternal age of 28.6 (5.9) years. SMM rates increased from 69.5 per 10 000 in 2012 to 79.7 per 10 000 in 2019 (rate difference [RD], 10.2; 95% CI, 5.8 to 14.6) without a significant change across the *ICD-10-CM/PCS* transition (RD, −3.2; 95% CI, −6.9 to 0.6). Of 20 SMM indicators, rates for 10 indicators significantly increased while 3 significantly decreased; 5 of these changes were associated with *ICD-10-CM/PCS* transition. Acute kidney failure had the largest increase, from 6.4 to 15.3 per 10 000 delivery hospitalizations (RD, 8.9; 95% CI, 7.5 to 10.3) with no change associated with *ICD* transition (RD, −0.1; 95% CI, −1.2 to 1.1). Disseminated intravascular coagulation had the largest decrease from 31.3 to 21.2 per 10 000 (RD, 10.2; 95% CI, −12.8 to −7.5), with a significant drop associated with *ICD* transition (RD, −7.9; 95% CI, −10.2 to −5.6). State SMM rates significantly decreased for 1 state and significantly increased for 21 states from 2012 to 2019 and associations with *ICD* transition varied.

**Conclusions and Relevance:**

In this cross-sectional study, overall US SMM rates increased from 2012 to 2019, which was not associated with the *ICD-10-CM/PCS* transition. However, data for certain indicators and states may not be comparable across coding systems; efforts are needed to understand SMM increases and state variation.

## Key Points

**Question:**

How did US rates of severe maternal morbidity (SMM), overall and by SMM indicator and state, change from 2012 to 2019 across the transition to *International Classification of Diseases, Tenth Revision, Clinical Modification/Procedure Coding System* (*ICD-10-CM/PCS*) coding?

**Findings:**

In this repeated cross-sectional analysis including 5.9 million delivery hospitalizations from 2012 to 2019, the national estimated rate of SMM (excluding blood transfusions) increased from 69.5 to 79.7 per 10 000 delivery hospitalizations, which was not associated with the *ICD-10-CM/PCS* transition. However, changes over time varied by indicator and state and some data may not be comparable across coding systems.

**Meaning:**

Overall SMM rates increased between 2012 and 2019, with noted variation by indicator and state.

## Teaser

This cross-sectional study analyzes trends in rates of severe maternal morbidity in US hospitals following the transition to the *International Classification of Diseases, Tenth Revision, Clinical Modification and Procedure Coding System* between 2012 and 2019.

## Introduction

Severe maternal morbidity (SMM), or unexpected life-threatening complications during delivery hospitalization, carries significant health and economic consequences.^1,2^ Over the past few decades, the US SMM rate has steadily increased through at least 2014.^3,4^ While the reasons for this rise are not fully known, the increasing prevalence of chronic conditions and other health risks among women prior to and during pregnancy (eg, hypertension, diabetes, and obesity) are likely contributors.^3^

Demonstrating its importance as a key maternal health indicator, SMM is a Healthy People 2030 objective^5^ and a National Outcome Measure for the Title V Maternal and Child Health Services State Block Grant Program administered by the Health Resources and Services Administration (HRSA).^6^ Surveillance of SMM, including the assessment of temporal trends and patterns, is critical for monitoring maternal health and evaluating clinical quality improvement efforts.^7^ However, surveillance using hospital discharge records has been complicated by a major coding transition and expansion from the ninth to the tenth revision of *International Classification of Disease Clinical Modification and Procedure Coding System (ICD-CM-PCS)* in October 2015.^8^ Using a segmented regression technique, a 2021 study^9^ of the Healthcare Cost and Utilization Project’s (HCUP) National Inpatient Sample (NIS) between 2012 and 2017 reported a significant decrease in the incidence of SMM at delivery immediately following the *ICD-10-CM/PCS* transition. However, recent code revisions^7,10^ were not incorporated and a longer follow-up time after transition may be necessary to examine potential delays in adaptation to the new coding system. Furthermore, the extent of state variation in SMM trends across the *ICD-10-CM/PCS* transition remained unknown.

The objectives of this study were to examine recent national and state trends in SMM rates in relation to *ICD-10-CM/PCS* transition using discharge data from the 2012 to 2019 NIS and State Inpatient Databases (SID).^11,12^ We used a combination of recommended approaches to examine *ICD-10-CM/PCS* transition,^13^ including bidirectional diagnosis and procedure code mapping and concordance tables, segmented regression, and graphic visualization.

## Methods

### Data Source and Study Population

National data were obtained from the 2012 to 2019 Agency for Healthcare Research and Quality’s (AHRQ) HCUP-NIS,^11^ an all-payer database of hospital discharge records—with the *ICD-10-CM/PCS* transition approximately centered in these years. Designed to be nationally representative, the NIS is a 20% systematic sample of all discharges from community, nonrehabilitation hospitals in participating states, which includes 48 states and the District of Columbia for at least 1 year of the study period. State data, examined by patient state of residence, were obtained from the SID for a total of 46 states and the District of Columbia, which contributed at least 1 year of data before and after *ICD-10-CM/PCS* transition. Alabama and Idaho were the only states that did not contribute any data during the study period, while Delaware and New Hampshire only contributed data after *ICD-10-CM/PCS* transition. Some gaps in data availability occurred for Alaska (2013-2014), Mississippi (2012), and the District of Columbia (2012) but data from these HCUP partners were able to be included by having at least some data before and after *ICD-10-CM/PCS* transition. Analyses were restricted to delivery hospitalizations identified by *ICD* diagnosis codes, Medicare Severity-Diagnosis Related Group (MS-DRG) delivery codes, and *ICD* procedure codes for selected delivery-related procedures.^14^ Per convention, analyses were further restricted to delivery hospitalizations for female individuals aged 12 to 55 years.^3,10,15^ As a secondary analysis of anonymized data, the AHRQ human protections administrator determined this project did not constitute research involving human participants; thus, informed consent and institutional review board approval were not required. The study follows the Strengthening the Reporting of Observational Studies in Epidemiology (STROBE) guidelines for reporting cross-sectional studies.

### Outcome Measures

At the national level, SMM was examined overall and for each included SMM indicator, as measured by diagnosis and procedure codes, in both *ICD-9-CM* (2012 to 2015 quarter 3) and *ICD-10-CM/PCS* (2015 quarter 4 to 2019) (eTable 1 in the Supplement). Consistent with recent federal SMM surveillance,^5,6^ blood transfusion was not included in the overall definition because transfusion alone without other indicators of SMM lacks specificity and lowers positive predictive value for “near miss” events.^16,17^ In-hospital mortality was examined as a secondary control outcome that is related to SMM but not captured by diagnosis or procedure codes, and thus unaffected by *ICD* version. At the state level, SMM was examined overall and for the most common SMM indicator.

### Statistical Analysis

Using AHRQ’s MapIT tool^18^ and Clinical Classifications Software Refined,^19^ we conducted bidirectional, forward (ie, from *ICD-9-CM* to *ICD-10-CM/PCS*) and backward (from *ICD-10-CM/PCS* to *ICD-9-CM*) conversion mapping to ensure consistent bridging and clinical concepts.^7,10^ Compared with previous US Centers for Disease Control and Prevention specifications, a total of 23 codes were added to *ICD-9-CM* and 83 codes were added to *ICD-10-CM/PCS* that were previously missed while 11 codes were dropped in *ICD-9-CM* and 16 codes were dropped in *ICD-10-CM/PCS* that were either conceptually inconsistent or implausible at delivery (eg, first trimester). In addition, shock codes involving sepsis and anesthesia were moved to their respective indicator categories as the primary cause. We also assigned code mapping types by indicator: 1-to-1 (exact), many-to-1 (detail reduction), 1-to-many (detail expansion), many-to-many (convoluted translation), and no translation (not present in both systems). These mapping types ranged from complete concordance to no concordance with the expectation that decreasing concordance would confer increasing likelihood of transition impact and inability to make comparisons across *ICD* versions.^13^

We calculated descriptive statistics for the patient characteristics of maternal age, self-reported race and ethnicity, primary expected payer, median zip code income quartile, and rural or urban residence based on a simplification of the National Center for Health Statistics’ urban-rural classification scheme for counties.^11,20^ For each outcome, we examined the overall change from 2012 to 2019 by comparing first and last estimates with absolute rate differences (RDs). We then determined change associated with *ICD* transition using a segmented regression approach common to both interrupted time series^21^ and sharp regression discontinuity designs.^22^ Used in other analyses of *ICD-10-CM* transition,^9,23,24,25^ the segmented regression model includes an immediate intercept change or so-called “jump” with *ICD* transition as well as a time trend or slope that is allowed to vary before and after the transition. The immediate change associated with *ICD* transition is captured through a dummy variable (0 for *ICD-9-CM*; 1 for *ICD-10-CM/PCS*). The trend was captured by a continuous quarterly time variable that was allowed to change across the *ICD* transition with a spline term (continuous variable with new baseline of 0 for the first data point in *ICD-10-CM*; 0 in *ICD-9-CM*). Consistent with regression discontinuity, the focus was on the immediate change associated with *ICD* transition controlling for underlying time trends, which accounted for longer-term changes in patient characteristics and care practices. Binomial regression models were implemented with an identity link for absolute contrasts with dummy variables for quarter to control for potential seasonality. We plotted residuals of aggregated quarterly data and no patterns were observed for time, indicating adequacy of the linear model. To promote comparability with the previous analysis of SMM across the *ICD* transition,^9^ we also restricted to the same set of years (2012 to 2017) with the only remaining difference being the revised SMM code set.

Using annual rates to promote stability, we examined and graphed SMM rates across the *ICD* transition to identify potential nonlinear trends (ie, changes preceding *ICD* transition) or anomalies (ie, 1- or 2-year outliers) that were inconsistent with a sustained transition-related impact. National estimates of rates, RDs, and 95% CIs were weighted and adjusted to account for the complex sampling design using the SVY command in Stata version 15.1 (StataCorp LLC). Statistical significance was determined using 2-sided tests and *P* < .05. No adjustments were made for multiple comparisons since these approaches increase type II error (false negatives) and there was an a priori basis for an association with *ICD* transition.^26^

## Results

At the national level, a total of 5 964 315 delivery hospitalizations from 2012 to 2019, representing a weighted total of 29.8 million deliveries, were included in the analysis, with a mean (SD) maternal age of 28.6 (5.9) years. Over half of all deliveries were to non-Hispanic White women (14 958 568 [50.2%]), those with a private primary expected payer (15 141 893 [50.8%]), and those who lived in large metropolitan areas (16 918 417 [56.7%]). There were no substantial changes in the distribution of patient characteristics across the *ICD-10-CM/PCS* transition (Table 1).

**Table 1.** Demographic Characteristics of Delivery Hospitalizations by Coding System, 2012-2019^a^

Demographic characteristic^b^	Pregnant individuals, Weighted No. (%)	
	*ICD-9-CM*: 2012-2015 Q3 (n = 14 165 784)	*ICD-10-CM/PCS*: 2015 Q4-2019 (n = 15 655 780)
Maternal age, mean (SD), y	28.2 (5.9)	28.9 (5.8)
Race and ethnicity		
Asian or Pacific Islander, non-Hispanic	748 630 (5.3)	931 964 (6.0)
Black, non-Hispanic	1 924 595 (13.6)	2 249 860 (14.4)
Hispanic	2 753 540 (19.4)	3 110 313 (19.9)
Other, non-Hispanic^c^	738 705 (5.2)	800 560 (5.1)
White, non-Hispanic	7 088 699 (50.0)	7 869 869 (50.3)
Unknown	911 615 (6.4)	693 214 (4.4)
Primary expected payer		
Medicaid	6 135 240 (43.4)	6 663 896 (42.6)
Other public	517 665 (3.7)	538 695 (3.5)
Private	7 120 019 (50.4)	8 021 874 (51.3)
Self-pay	369 000 (2.6)	412 435 (2.6)
Zip code income quartile^d^		
1 (lowest)	3 939 660 (28.3)	4 367 722 (28.2)
2	3 506 584 (25.2)	3 895 547 (25.1)
3	3 440 630 (24.7)	3 845 917 (24.8)
4 (highest)	3 058 375 (21.9)	3 398 738 (21.9)
Urban/rural residence^e^		
Large metropolitan	7 991 681 (56.6)	8 926 736 (57.2)
Small metropolitan	4 130 080 (29.2)	4 590 513 (29.4)
Nonmetropolitan	2 007 093 (14.2)	2 097 091 (13.4)

Abbreviation: *ICD-10-CM/PCS*, *International Classification of Diseases, 10th Revision, Clinical Modification and Procedure Coding System*.

^a^ Data are from the Agency for Healthcare Research and Quality, Healthcare Cost and Utilization Project, National Inpatient Sample (HCUP-NIS), 2012-2019.^11^

^b^ All characteristics were missing ≤1% of data with the exception of race and ethnicity, where missing data are presented as an unknown category.

^c^ Includes American Indian or Alaska Native, other race, and more than 1 race.

^d^ Median household income of residents in the patient’s zip code was updated annually; 2019 range for quartile 1 was less than $48 000; quartile 2, $48 000 to $60 999; quartile 3, $61 000 to $81 999; and quartile 4, at least $82 000.

^e^ Based on a simplification of the National Center for Health Statistics Urban Rural Classification Scheme to the following categories: large metropolitan counties (≥1 million population), small metropolitan counties (50 000-999 999 population), and nonmetropolitan counties.

Overall, the number of SMM diagnosis and procedure codes (excluding blood transfusion) expanded from 244 in *ICD-9-CM* to 437 in *ICD-10-CM/PCS*. Among the 20 included SMM indicators, 2 had a 1-to-1 translation, 1 had a many-to-1 translation, 12 had a 1-to-many translation, and 5 had a many-to-many translation (Table 2).

**Table 2.** Number of Codes and Mapping Type by Severe Maternal Morbidity Indicator, *ICD-9-CM* and *ICD-10-CM/PCS*

Severe maternal morbidity indicator	Delivery hospitalizations, No.		Mapping type
	*ICD-9-CM* codes (n = 244)	*ICD-10-CM/PCS* codes (n = 437)	
Acute myocardial infarction (DX)	30	17	Many to many
Acute kidney failure (DX)	8	6	Many to 1
Adult respiratory distress syndrome (DX)	7	17	1 to many
Air and thrombotic embolism (DX)	25	29	1 to many
Amniotic fluid embolism (DX)	5	5	1 to 1
Aneurysm (DX)	12	13	1 to many
Cardiac arrest/ventricular fibrillation (DX)	3	5	1 to many
Conversion of cardiac rhythm (PR)	6	2	Many to many
Disseminated intravascular coagulation (DX)	8	29	1 to many
Eclampsia (DX)	5	6	1 to many
Heart failure/arrest during surgery (DX)	1	6	1 to many
Hysterectomy (PR)	6	4	Many to many
Puerperal cerebrovascular disorders (DX)	54	198	Many to many
Pulmonary edema/acute heart failure (DX)	13	20	1 to many
Sepsis (DX)	23	27	1 to many
Severe anesthesia complications (DX)	17	25	1 to many
Shock (DX)	12	10	Many to many
Sickle cell disease with crisis (DX)	5	12	1 to many
Temporary tracheostomy (PR)	1	3	1 to many
Ventilation (PR)	3	3	1 to 1

Abbreviations: DX, diagnosis; *ICD-9-CM*, *International Classification of Disease, 9th Revision, Clinical Modification*; *ICD-10-CM/PCS*, *International Classification of Diseases, 10th Revision, Clinical Modification and Procedure Coding System*; PR, procedure.

**Figure 1.**
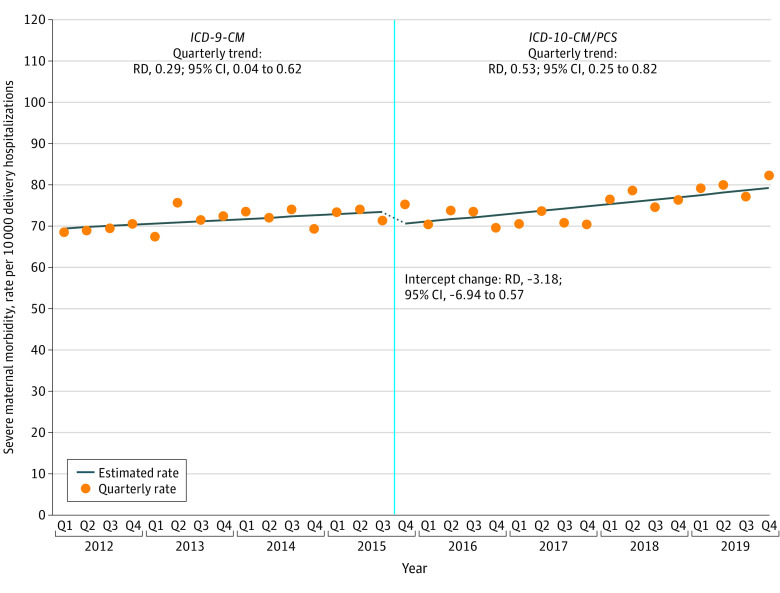
Severe Maternal Morbidity per 10 000 Delivery Hospitalizations, 2012-2019 Abbreviations: *ICD-9-CM*, *International Classification of Disease, 9th Revision, Clinical Modification*; *ICD-10-CM/PCS*, *International Classification of Diseases, 10th Revision, Clinical Modification and Procedure Coding System*; RD, rate difference. Estimated rates were obtained from segmented linear binomial regression models including the *ICD-10-CM* transition, quarterly time trends (allowed to vary before and after transition), and quarter control variables. Data are from the Agency for Healthcare Research and Quality, Healthcare Cost and Utilization Project, National Inpatient Sample (HCUP-NIS), 2012-2019.^11^

Between 2012 and 2019, SMM rates increased from 69.5 to 79.7 per 10 000 delivery hospitalizations (RD, 10.2; 95% CI, 5.8 to 14.6) without a statistically significant change across the *ICD-10-CM/PCS* transition (RD, −3.2; 95% CI, −6.9 to 0.6) (Figure 1; Table 3). When restricted to the same set of years as a prior study (2012 to 2017), there was also no significant change associated with *ICD-10-CM/PCS* transition (RD, 0; 95% CI, −4.1 to 4.1). Compared with 2012, annual SMM rates were only significantly higher for 2018 and 2019. In-hospital mortality per 100 000 delivery hospitalizations did not increase significantly over the study period (RD, 1.7; 95% CI, −0.5 to 4.0), and there was no change associated with *ICD-10-CM/PCS* transition (RD, 1.5; 95% CI, −0.9 to 3.9).

**Table 3.** Rates of Severe Maternal Morbidity and In-Hospital Mortality Among Delivery Hospitalizations, 2012-2019^a^

Characteristic	Year, Rate per 10 000 delivery hospitalizations								Rate difference (95% CI)	
	2012	2013	2014	2015 Q1-Q3^b^	2016	2017	2018	2019	Total change, 2019 vs 2012^c^	Change with *ICD-10-CM/PCS*^d^
Severe maternal morbidity, weighted No.	26 185	26 910	27 520	20 890	27 260	26 495	27 860	28 570		
Severe maternal morbidity	69.5	71.9	72.4	73.1	72.0	71.5	76.7	79.7	10.2 (5.8 to 14.6)	−3.2 (−6.9 to 0.6)
Indicator^e^										
Acute myocardial infarction	0.2	0.3	0.1	0.2	0.4	0.3	0.4	0.5	0.2 (0.1 to 0.4)	0.2 (0.0 to 0.3)
Aneurysm	0.3	0.2	0.3	0.4	0.2	0.3	0.4	0.4	0.1 (−0.1 to 0.3)	0.0 (−0.2 to 0.1)
Acute kidney failure	6.4	8.1	8.3	8.6	10.3	11.5	13.0	15.3	8.9 (7.5 to 10.3)	−0.1 (−1.2 to 1.1)
Adult respiratory distress syndrome	6.9	7.6	8.7	8.1	8.7	9.2	10.2	10.1	3.3 (2.3 to 4.3)	−0.8 (−1.8 to 0.2)
Amniotic fluid embolism	0.4	0.5	0.4	0.4	0.5	0.6	0.6	0.6	0.2 (−0.0 to 0.5)	0.1 (−0.1 to 0.4)
Cardiac arrest/ventricular fibrillation	0.6	0.7	0.9	0.9	0.9	0.9	1.2	1.0	0.4 (0.1 to 0.7)	−0.2 (−0.5 to 0.1)
Conversion of cardiac rhythm	0.7	0.7	0.8	0.7	0.8	0.9	0.9	1.0	0.3 (0.0 to 0.6)	0.0 (−0.3 to 0.3)
Disseminated intravascular coagulation	31.3	31.6	29.4	28.2	19.8	20.3	20.9	21.2	−10.2 (−12.8 to −7.5)	−7.9 (−10.2 to −5.6)
Eclampsia	6.9	6.6	6.6	6.6	10.4	7.9	7.0	6.9	0.0 (−0.9 to 0.9)	4.3 (3.4 to 5.3)
Heart failure/arrest during surgery	1.1	0.9	0.8	0.8	0.1	0.1	0.1	0.1	−1.0 (−1.3 to −0.8)	−0.6 (−0.8 to −0.4)
Puerperal cerebrovascular disorders	3.6	3.7	3.6	3.8	2.9	2.8	3.4	3.5	−0.1 (−0.7 to 0.5)	−1.0 (−1.6 to −0.4)
Pulmonary edema/acute heart failure	4.0	4.6	4.6	4.7	6.1	6.2	6.6	7.3	3.2 (2.4 to 4.1)	1.0 (0.2 to 1.8)
Severe anesthesia complications	1.2	1.5	1.2	1.4	0.8	0.6	0.7	0.8	−0.4 (−0.7 to −0.1)	−0.7 (−1.0 to −0.3)
Sepsis	4.4	4.9	6.9	7.5	9.0	9.4	10.6	11.1	6.8 (5.3 to 8.2)	0.2 (−0.9 to 1.3)
Shock	3.4	4.1	4.3	4.8	5.7	5.5	6.8	6.9	3.5 (2.8 to 4.3)	0.2 (−0.6 to 0.9)
Sickle cell disease with crisis	1.1	0.9	1.0	1.1	1.2	1.3	1.3	1.1	0.0 (−0.3 to 0.4)	0.2 (−0.2 to 0.6)
Air and thrombotic embolism	2.0	1.9	2.2	2.2	3.1	3.0	3.6	3.0	1.0 (0.4 to 1.5)	1.2 (0.6 to 1.7)
Hysterectomy	9.6	10.5	10.6	11.5	11.6	11.6	12.7	12.5	2.9 (1.6 to 4.1)	−0.3 (−1.5 to 0.9)
Temporary tracheostomy	0.2	0.1	0.3	0.3	0.2	0.2	0.2	0.2	0.0 (−0.2 to 0.2)	−0.1 (−0.2 to 0.1)
Ventilation	4.8	5.2	5.9	4.8	4.9	4.8	5.2	4.7	−0.1 (−0.8 to 0.7)	−0.4 (−1.2 to 0.3)
In-hospital mortality	3.9	5.1	5.5	3.5	6.0	6.1	7.0	5.6	1.7 (−0.5 to 4.0)	1.5 (−0.9 to 3.9)

Abbreviations: *ICD-9-CM*, *International Classification of Disease, 9th Revision, Clinical Modification*; *ICD-10-CM/PCS*, *International Classification of Disease, 10th Revision, Clinical Modification and Procedure Coding System*.

^a^ Data are from the Agency for Healthcare Research and Quality, Healthcare Cost and Utilization Project, National Inpatient Sample (HCUP-NIS), 2012-2019.^11^

^b^ Excludes the fourth quarter, to distinguish the transition to *ICD-10-CM* in October 2015.

^c^ Total change is the absolute rate difference (2019 minus 2012).

^d^ Change with *ICD-10-CM/PCS* is the immediate change associated with transition obtained from segmented linear binomial regression models controlling for quarter and quarterly time trends allowed to vary before and after transition.

^e^ Follows indicator numbering used by US Centers for Disease Control and Prevention; blood transfusion is excluded because of poor positive predictive value in the absence of other indicators.

In 2019, the top 5 indicators of SMM included disseminated intravascular coagulation (DIC) (21.2 per 10 000 hospitalizations), acute kidney failure (15.3 per 10 000 hospitalizations), hysterectomy (12.5 per 10 000 hospitalizations), sepsis (11.1 per 10 000 hospitalizations), and adult respiratory distress syndrome (10.1 per 10 000 hospitalizations) similar to the top 5 in 2012 with the exception of sepsis, which ranked seventh and was superseded by eclampsia (2012: sepsis, 4.4 cases per 10 000 hospitalizations vs eclampsia, 6.9 cases per 10 000 hospitalizations) (Table 3). From 2012 to 2019, rates of 10 SMM indicators increased significantly, ranging from 0.2 per 10 000 delivery hospitalizations for acute myocardial infarction (95% CI, 0.1 to 0.4) to 8.9 per 10 000 for acute kidney failure (95% CI, 7.5 to 10.3). Of the 10 indicators with increases from 2012 to 2019, only 2 were associated with *ICD-10-CM/PCS* transition: pulmonary edema and/or acute heart failure increased by 3.2 per 10 000 delivery hospitalizations overall (95% CI, 2.4 to 4.1) with 1.0 per 10 000 associated with *ICD-10-CM/PCS* (95% CI, 0.2 to 1.8), while the entirety of the increase for air and thrombotic embolism (RD, 1.2; 95% CI, 0.6 to 1.7) was associated with *ICD-10-CM/PCS*. There were significant decreases in 3 SMM indicators which were all associated with *ICD-10-CM/PCS* transition: DIC (RD, −7.9; 95% CI, −10.2 to −5.6), heart failure or arrest during surgery (RD, −0.6; 95% CI, −0.8 to −0.4), and severe anesthesia complications (RD, −0.7; 95% CI, −1.0 to −0.3). Both eclampsia and puerperal cerebrovascular disorders had changes associated with *ICD-10-CM/PCS* transition, an increase and decrease respectively, yet lacked overall change from 2012 to 2019 and had 1 or 2 year anomalies following the transition that were not consistent with a sustained impact.

**Figure 2.**
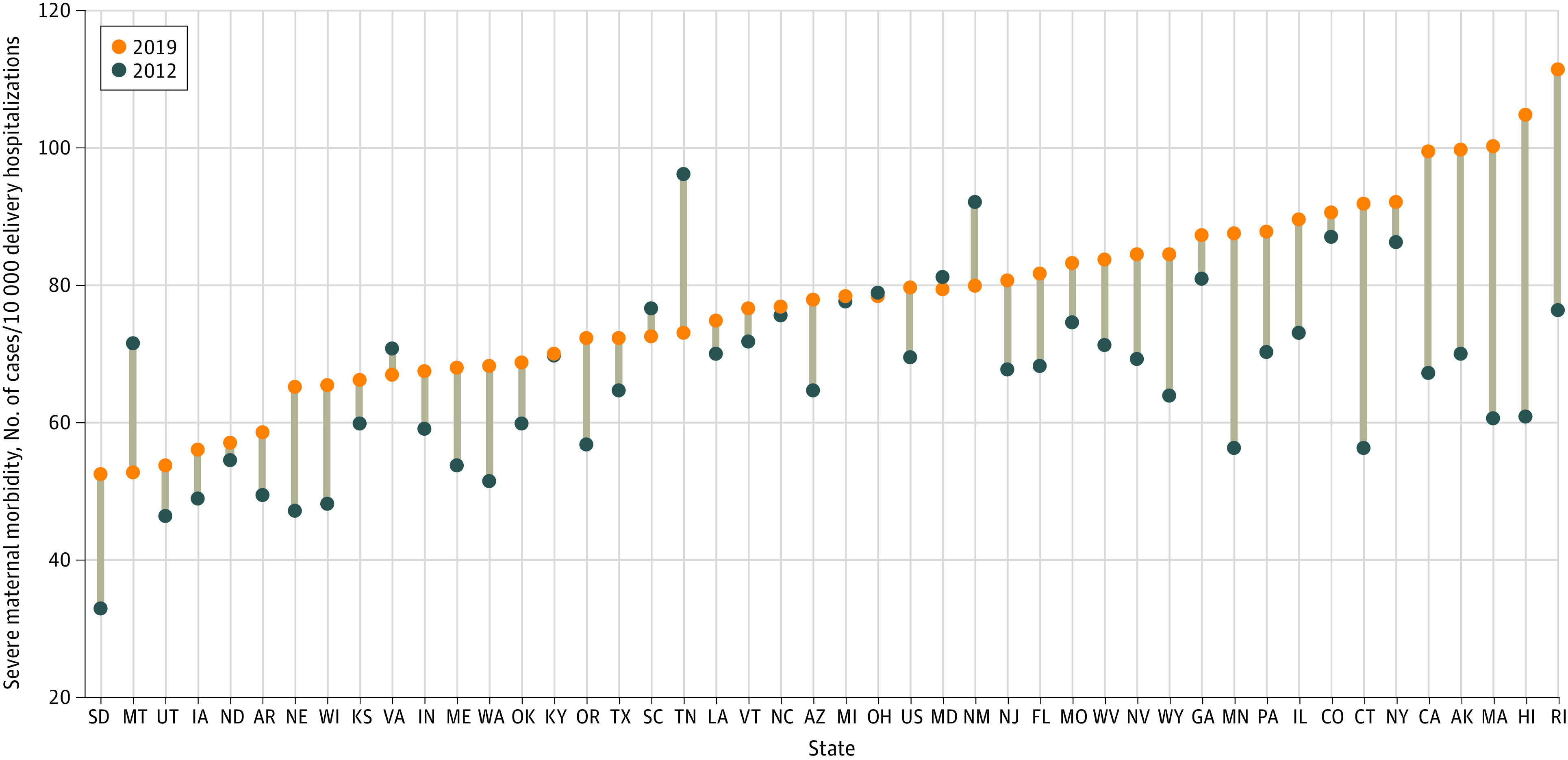
Severe Maternal Morbidity per 10 000 Delivery Hospitalizations by State, 2012 and 2019 Data are from the Agency for Healthcare Research and Quality, Healthcare Cost and Utilization Project, National Inpatient Sample, and State Inpatient Databases, 2012 and 2019.^11^

At the state level, SMM rates ranged from 52.5 per 10 000 delivery hospitalizations in South Dakota to 111.3 per 10 000 delivery hospitalizations in Rhode Island in 2019 (Figure 2; eTable 2 in the Supplement). Many of the states with the highest rates in 2019 had substantial increases since 2012. For example, 6 of the top 10 states had increases of approximately 30 per 10 000 or more, which was triple that of the national increase. SMM rates significantly increased for 21 states between 2012 and 2019, ranging from 5.9 per 10 000 delivery hospitalizations in New York to 44.0 per 10 000 in Hawaii. Only 3 state increases were associated with *ICD-10-CM/PCS* transition (Massachusetts, Rhode Island, and Wisconsin). Only 1 state (Tennessee) had a significant decrease over the study period (RD, −23.0; 95% CI, −32.7 to −13.2), which was associated with the *ICD-10-CM/PCS* transition (RD, −14.7; 95% CI, −25.0 to −4.4). Further analysis of DIC, which had the largest national decrease associated with *ICD-10-CM/PCS* transition, also showed state variability, with 1 of 4 state DIC increases associated with the coding transition and 17 of 24 state DIC decreases associated with the coding transition (eTable 3 in the Supplement).

## Discussion

Overall, the national rate of SMM increased by 10.2 per 10 000 delivery hospitalizations, or 15%, from 2012 to 2019, with the largest increases attributable to acute kidney failure and sepsis. Although these increases were not associated with transition to *ICD-10-CM/PCS*, several indicators had changes associated with *ICD* transition. DIC decreased by 10.2 per 10 000 delivery hospitalizations, or nearly a third over the study period, with most of this drop associated with *ICD-10-CM/PCS* transition. Other indicator-level changes associated with *ICD* transition were smaller in magnitude but included a decrease for severe anesthesia complications, an increase in air and thrombotic embolism, and counterbalancing changes in heart failure during surgery (a decrease) and pulmonary edema/acute heart failure (an increase). At the state level, nearly half of all states had significant increases in SMM rates from 2012 to 2019 that were generally not associated with *ICD* transition, mirroring the national data. However, half of all states lacked statistically significant SMM rate changes with 1 significant decrease also observed.

Previous studies of *ICD-9-CM* data showed increasing rates of SMM, with or without blood transfusion, through 2014 or 2015.^1,3,4^ Our study of SMM, without including blood transfusion, found continued increases in *ICD-10-CM/PCS* through 2019 reaching a rate of nearly 80 per 10 000 delivery hospitalizations. Although SMM is still a rare event, affecting less than 1% of delivery hospitalizations, it is approximately 40 times as common as maternal mortality,^27^ with nearly 30 000 annual cases carrying significant medical costs,^2^ trauma, and long-term rehabilitation.^1^ Our findings of overall increases and comparability of SMM rates across the *ICD-10-CM/PCS* transition conflict with a prior study using an older SMM algorithm that showed a significant decrease of 6 per 10 000 delivery hospitalizations and a reversal of trends.^9^ Overall increases in our study were confined to 2018 and 2019, 2 years not included in the previous analysis. Our analysis also used a revised code set achieved through careful bidirectional diagnosis and procedure code mapping and translation that improved ascertainment in *ICD-10-CM/PCS*.^10^ When restricted to the same set of years as the prior study (2012 to 2017), there was still no change associated with *ICD-10-CM/PCS* transition, indicating that the revised code set helped to bridge across coding systems.

Despite careful code revision, certain indicators remained affected in both directions with the largest change—a drop for DIC—that was consistent with the previous study. Significant differences in either direction are not surprising given the complexity of code translation, including both increases and decreases in code specificity and detail in *ICD-10-CM/PCS*. A small study of dual-coded discharges in 2 university hospitals showed comparability ratios for *ICD-10-CM/PCS* vs *ICD-9-CM* that were both above and below unity.^28^ Studies of other outcomes, including birth defects,^23^ injury,^24^ and opioid-related diagnoses,^25^ have also shown variability across indicators in *ICD* transition impact. In addition, the SMM indicators with rate changes associated with *ICD* transition were among those identified as having lower positive predictive value in a comparison of *ICD-9-CM* discharge codes vs expert clinical review of medical records.^29^ While overall SMM has reasonable positive predictive value, especially when blood transfusion is excluded,^16,17,29^ certain indicators that involve more clinical judgment and information to establish severity or determine peripartum onset may be less reliably coded in administrative discharge records.^29^

To our knowledge, this is the first study to examine state variation in SMM rates, revealing variability in overall changes and associations with the *ICD-10-CM/PCS* transition. State differences in the association of the transition on SMM may reflect variation in coding quality and accuracy as well as different relative proportions or case mix of SMM indicators. However, state variation in rate changes associated with *ICD* transition persisted when restricted to the same indicator (ie, DIC), confirming a role for variability in data quality and documentation. Over the 8 years of the study, 7 states had dramatic SMM rate increases at least triple the national increase (Alaska, California, Connecticut, Hawaii, Massachusetts, Minnesota, Rhode Island). An earlier study of California data from 2008 to 2014 found that SMM increases (including blood transfusion) could not be explained by changes in a variety of risk factors, including maternal age, prepregnancy comorbidities, and cesarean delivery.^30^ The national increase appears to have occurred mainly for metropolitan residents delivering in high volume hospitals,^15^ raising the possibility of improved diagnostic ascertainment through quality improvement activities^7,31^ rather than real increases. In addition, SMM indicators with lower positive predictive value^16,29^ may be contributing to increases and could suggest a need for measure refinement. The lack of concomitant increases in in-hospital mortality may support improved SMM ascertainment or increases in less severe or false positive cases. Understanding SMM trends and state variation represent important avenues for future research.

### Limitations

This study had several limitations. The main analytic limitation is the possibility that unrelated contemporaneous changes may explain observed associations with the *ICD-10-CM/PCS* transition. However, examination of annual estimates show that changes associated with *ICD* transition were dramatic and inconsistent with any other single year changes. The credibility of results is also supported by the absence of *ICD* associations for the 2 indicators with 1-to-1 translation as well as the control outcome of in-hospital mortality that should be unaffected by *ICD* coding. Longer-term trends in patient characteristics and care practices were controlled for with time variables and potential seasonality was controlled for with quarterly variables, with multiple years of data after the transition to allow for potential delays in adaptation. While overall trends are truncated because of substantial data lags, continuing rises through 2021 are likely with increased maternal morbidity associated with COVID-19.^32,33^ Furthermore, HCUP does not include and is not generalizable to federal facilities, such as military and Indian Health Service hospitals. By design, this study was restricted to delivery hospitalizations in the absence of linked data to identify postpartum readmissions but approximately 15% of SMM cases occur after delivery discharge.^34^ Thus, the total burden and incidence of SMM is understated with trends across the *ICD-10-CM/PCS* transition in postdischarge SMM not known.

## Conclusions

In this cross-sectional study, overall US SMM rates, excluding blood transfusion, increased from 2012 to 2019, which was not associated with the *ICD-10-CM/PCS* transition. However, data for certain SMM indicators and for certain states were associated with the coding transition and may not be comparable across coding systems. Efforts are needed to better understand recent SMM increases and state-level SMM variation.

## Notes

Supplement. **eTable 1.** Severe Maternal Morbidity Code Detail and Mapping Type by Indicator, *ICD-9-CM* and *ICD-10-CM/PCS* **eTable 2.** Severe Maternal Morbidity per 10 000 Delivery Hospitalizations by State, 2012-2019 **eTable 3.** Disseminated Intravascular Coagulation per 10 000 Delivery Hospitalizations by State, 2012-2019
