# Supplementary material for: Trends in Severe Maternal Morbidity in the US Across the Transition to ICD-10-CM/PCS From 2012-2019
Source: JAMA Netw Open. 2022 Jul 28;5(7):e2222966. doi: 10.1001/jamanetworkopen.2022.22966 (PMC9335134; doi:10.1001/jamanetworkopen.2022.22966)
Supplement: Supplement. — eTable 1. Severe Maternal Morbidity Code Detail and Mapping Type by Indicator, ICD-9-CM and ICD-10-CM/PCS eTable 2. Severe Maternal Morbidity per 10 000 Delivery Hospitalizations by State, 2012-2019 eTable 3. Disseminated Intravascular Coagulation per 10 000 Delivery Hospitalizations by State, 2012-2019 [file jamanetwopen-e2222966-s001.pdf]

Supplemental Online Content

Hirai AH, Owens PL, Reid LD, Vladutiu CJ, Main EK. Trends in severe maternal morbidity in the US across the transition to *ICD-10-CM/PCS* from 2012-2019. *JAMA Netw Open.* 2022;5(7):e2222966. doi:10.1001/jamanetworkopen.2022.22966

eTable 1. Severe Maternal Morbidity Code Detail and Mapping Type by Indicator, *ICD-9-CM* and *ICD-10-CM/PCS*

eTable 2. Severe Maternal Morbidity per 10 000 Delivery Hospitalizations by State, 2012-2019

eTable 3. Disseminated Intravascular Coagulation per 10 000 Delivery Hospitalizations by State, 2012-2019

This supplemental material has been provided by the authors to give readers additional information about their work.

© 2022 Hirai AH et al. *JAMA Network Open.*

eTable 1. Severe Maternal Morbidity Code Detail and Mapping Type by Indicator, *ICD-9-CM* and *ICD-10-CM/PCS*

Severe Maternal Morbidity Indicator	ICD-9-CM Codes (Description)	ICD-10-CM/PCS Codes (Description)
**1. Acute myocardial infarction (DX)**	410.0x-410.5x (AMI of anterolateral, other anterior, inferolateral, inferoposterior, other inferior, other lateral wall)	I21.xx (Acute myocardial infarction)
	410.6x (True posterior wall infarction)	I22.x (Subsequent ST elevation (STEMI) and non-ST elevation (NSTEMI) myocardial infarction)
	410.7x (Subendocardial infarction)	
	410.8x (Acute myocardial infarction of other specified sites)	
	410.9x (Acute myocardial infarction of unspecified site)	
**2. Aneurysm (DX)**	441.0x (Dissection of aorta)	I71.xx (Aortic aneurysm and dissection)
	441.1, 441.2 (Thoracic aneurysm)	I79.0 (Aneurysm of aorta in diseases classified elsewhere)
	441.3, 441.4 (Abdominal aneurysm)	
	441.5, 441.9 (Aortic aneurysm)	
	441.6, 441.7 (Thoracoabdominal aneurysm)	
**3. Acute renal failure (DX)**	584.5 - 584.9 (Acute kidney failure)	N17.x (Acute kidney failure)
	669.3x (Acute kidney failure following labor and delivery)	O90.4 (Postpartum acute kidney failure)
**4. Adult respiratory distress syndrome (DX)**	518.5x (Acute respiratory failure, acute and chronic, other pumonary insufficiency following trauma and surgery)	J80 (Acute respiratory distress syndrome)
	518.81 (Acute respiratory failure)	J95.1 (Acute pulmonary insufficiency following thoracic surgery)
	518.82 (Other pulmonary insufficiency, not elsewhere classified)	J95.2 (Acute pulmonary insufficiency following nonthoracic surgery)
	518.84 (Acute and chronic respiratory failure)	J95.3 (Chronic pulmonary insufficiency following surgery)
	799.1 (Respiratory arrest)	J95.82x (Postprocedural respiratory failure)
		J96.0x (Acute respiratory failure)
		J96.2x (Acute and chronic respiratory failure)
		J96.9x (Respiratory failure, unspecified)
		R06.03 (Acute respiratory distress syndrome)
		R09.2 (Respiratory arrest)
**5. Amniotic fluid embolism (DX)**	673.10 (Amniotic fluid embolism, unspecified as to episode of care or not applicable)	O88.112 (Amniotic fluid embolism in pregnancy, second trimester)
	673.11 (Amniotic fluid embolism, delivered, with or without mention of antepartum condition)	O88.113 (Amniotic fluid embolism in pregnancy, third trimester)
	673.12 (Amniotic fluid embolism, delivered, with mention of postpartum complication)	O88.119 (Amniotic fluid embolism in pregnancy, unspecified trimester)
	673.13 (Amniotic fluid embolism, antepartum condition or complication)	O88.12 (Amniotic fluid embolism in childbirth)

© 2022 Hirai AH et al. *JAMA Network Open.*

Severe Maternal Morbidity Indicator	ICD-9-CM Codes (Description)	ICD-10-CM/PCS Codes (Description)
	673.14 (Amniotic fluid embolism, postpartum condition or complication)	O88.13 (Amniotic fluid embolism in the puerperium)
**6. Cardiac arrest/ventricular fibrillation (DX)**	427.41 (Ventricular fibrillation)	I46.x (Cardiac arrest)
	427.42 (Ventricular flutter)	I49.01 (Ventricular fibrillation)
	427.5 (Cardiac arrest)	I49.02 (Ventricular flutter)
**7. Conversion of cardiac rhythm (PR)**	99.60 (Cardiopulmonary resuscitation, not otherwise specified)	5A12012 (Performance of cardiac output, single, manual)
	99.61 (Atrial cardioversion)	5A2204Z (Restoration of cardiac rhythm, single)
	99.62 (Other electric countershock of heart)	
	99.63 (Closed chest cardiac massage)	
	99.64 (Carotid sinus stimulation)	
	99.69 (Other conversion of cardiac rhythm)	
**8. Disseminated intravascular coagulation (DX)**	286.6 (Defibrination syndrome)	D65 (Disseminated intravascular coagulation [defibrination syndrome])
	286.9 (Other and unspecified coagulation defects)	D68.8 (Other specified coagulation effects)
	641.3x (Antepartum hemorrhage associated with coagulation defects)	D68.9 (Coagulation defect, unspecified)
	666.3x (Postpartum coagulation defects)	O45.002, O45.003, O45.009 (Premature separation of placenta with coagulation defect, unspecified; second, third, unspecified trimester)
		O45.012, O45.013, O45.019 (Premature separation of placenta with afibrinogenemi; second, third, unspecified trimester)
		O45.022, O45.023, O45.029 (Premature separation of placenta with disseminated intravascular coagulation; second, third, unspecified trimester)
		O45.092, O45.093, O45.099 (Premature separation of placenta with other coagulation defect; second, third, unspecified trimester)
		O46.002, O46.003, O46.009 (Antepartum hemorrhage with coagulation defect, unspecified; second, third, unspecified trimester)
		O46.012, O46.013, O46.019 (Antepartum hemorrhage with afibrinogenemia; second, third, unspecified trimester)
		O46.022, O46.023, O46.029 (Antepartum hemorrhage with disseminated intravascular coagulation; second, third, unspecified trimester)
		O46.092, O46.093, O46.099 (Antepartum hemorrhage with other coagulation defect; second, third, unspecified trimester)
		O67.0 (Intrapartum hemorrhage with coagulation defect)
		O72.3 (Postpartum coagulation defects)
**9. Eclampsia (DX)**	642.60 (Eclampsia, unspecified as to episode of care or not applicable)	O15.00, O15.02, O15.03 (Eclampsia complicating pregnancy; second, third, unspecified trimester)

© 2022 Hirai AH et al. *JAMA Network Open.*

Severe Maternal Morbidity Indicator	ICD-9-CM Codes (Description)	ICD-10-CM/PCS Codes (Description)
	641.61, 642.62 (Eclampsia, delivered, with or without mention of antepartum or postpartum)	O15.1 (Eclampsia complicating childbirth)
	642.63 (Eclampsia, antepartum condition or complication)	O15.2 (Eclampsia complicating the puerperium)
	642.64 (Eclampsia, postpartum condition or complication)	O15.9 (Eclampsia, unspecified as to time period)
**10. Heart failure/arrest during surgery or procedure (DX)**	997.1 (Cardiac complications, not elsewhere classified)	I97.12x (Postprocedural cardiac arrest)
		I97.13x (Postprocedural heart failure)
		I97.710 (Intraoperative cardiac arrest during cardiac surgery)
		I97.711 (Intraoperative cardiac arrest during other surgery)
**11. Puerperal cerebrovascular disorders (DX)**	046.3 (Progressive multifocal leukoencephalopathy)	A81.2 (Progressive multifocal leukoencephalopathy)
	348.39 (Other encephalopathy)	G45.x (Transient cerebral ischemic attacks and related syndromes)
	362.34 (Transient retinal arterial occlusion)	G46.x (Vascular syndromes of brain in cerebrovascular diseases)
	430 (Subarachnoid hemorrhage)	G93.49 (Other encephalopathy)
	431 (Intracerebral hemorrhage)	H34.0x (Transient retinal artery occlusion)
	432.x (Intracranial hemorrhage)	I60.xx (Nontraumatic subarachnoid hemorrhage)
	433.xx (Occlusion and stenosis of artery)	I61.xx (Nontraumatic intracerebral hemorrhage)
	434.xx (Cerebral thrombosis, embolism, or occlusion)	I62.xx (Other and unspecified nontraumatic intracranial hemorrhage)
	435.x (Transient cerebral ischemia)	I63.xx (Cerebral infarction)
	436 (Acute, but ill-defined, cerebrovascular disease)	I65.xx (Occlusion and stenosis of precerebral arteries, not resulting in cerebral infarction)
	437.x (Other and Unspecified cerebrovascular disease)	I66.xx (Occlusion and stenosis of cerebral arteries, not resulting in cerebral infarction)
	671.5x (Other phlebitis and thrombosis complicating pregnancy and the puerperium)	I67.xx (Other cerebrovascular diseases)
	674.0x (Cerebrovascular disorders in the puerperium)	I68.xx (Cerebrovascular disorders in diseases classified elsewhere)
	997.02 (Iatrogenic cerebrovascular infarction or hemorrhage)	I97.81x (Intraoperative cerebrovascular infarction)
		I97.82x (Postprocedural cerebrovascular infarction)
		O22.50, O22.52, O22.53 (Cerebral venous thrombosis in pregnancy; unspecified, second, third trimester)
		O87.3 (Cerebral venous thrombosis in the puerperium)
**12. Pulmonary edemaand Acute heart failure (DX)**	518.4 (Acute edema of lung, unspecified)	J81.0 (Acute pulmonary edema)
	428 (Congestive heart failure, unspecified)	I50.1 (Left ventricular failure)
	428.1 (Left heart failure)	I50.20 (Unspecified systolic (congestive) heart failure)
	428.2 (Systolic heart failure, unspecified)	I50.21 (Acute systolic (congestive) heart failure)
	428.21 (Acute systolic heart failure)	I50.23 (Acute on chronic systolic (congestive) heart failure)

© 2022 Hirai AH et al. *JAMA Network Open.*

Severe Maternal Morbidity Indicator	ICD-9-CM Codes (Description)	ICD-10-CM/PCS Codes (Description)
	428.23 (Acute on chronic systolic heart failure)	I50.30 (Unspecified diastolic (congestive) heart failure)
	428.3 (Diastolic heart failure, unspecified)	I50.31 (Acute diastolic (congestive) heart failure)
	428.31 (Acute diastolic heart failure)	I50.33 (Acute on chronic diastolic (congestive) heart failure)
	428.33 (Acute on chronic diastolic heart failure)	I50.40 (Unspecified combined systolic (congestive) and diastolic (congestive) heart failure)
	428.4 (Combined systolic and diastolic heart failure, unspecified)	I50.41 (Acute combined systolic (congestive) and diastolic (congestive) heart failure)
	428.41 (Acute combined systolic and diastolic heart failure)	I50.43 (Acute on chronic combined systolic (congestive) and diastolic (congestive) heart failure)
	428.43 (Acute on chronic combined systolic and diastolic heart failure)	I50.810 (Right heart failure, unspecified)
	428.9 (Heart failure, unspecified)	I50.811 (Acute right heart failure)
		I50.813 (Acute on chronic right heart failure)
		I50.814 (Right heart failure due to left heart failure)
		I50.82 (Biventricular heart failure)
		I50.83 (High output heart failure)
		I50.84 (End stage heart failure)
		I50.89 (Other heart failure)
		I50.9 (Heart failure, unspecified)
**13. Severe anesthesia complications (DX)**	668.0x (Pulmonary complications of anesthesia or other sedation in labor and delivery)	O29.112, O29.113, O29.119 (Cardiac arrest due to anesthesia during pregnancy; second, third, unspecified trimester)
	668.1x (Cardiac complications of anesthesia or other sedation in labor and delivery)	O29.122, O29.123, O29.129 (Cardiac failure due to anesthesia during pregnancy; second, third, unspecified trimester)
	668.2x (Central nervous system complications of anesthesia or other sedation in labor and delivery)	O29.192, O29.193, O29.199 (Oth cardiac complications of anesthesia during pregnancy; second, third, unspecified trimester)
	995.4 (Shock due to anesthesia, not elsewhere classified)	O29.212, O29.213, O29.219 (Cerebral anoxia due to anesthesia during pregnancy; second, third, unspecified trimester)
	995.86 (Malignant hyperthermia)	O29.292, O29.293, O29.299 (Other central nervous system complications of anesthesia during pregnancy; second, third, unspecified trimester)
		O74.0 (Aspiration pneumonitis due to anesthesia during labor and delivery)
		O74.1 (Other pulmonary complications of anesthesia during labor and delivery)
		O74.2 (Cardiac complications of anesthesia during labor and delivery)
		O74.3 (Central nervous system complications of anesthesia during labor and delivery)

© 2022 Hirai AH et al. *JAMA Network Open.*

Severe Maternal Morbidity Indicator	ICD-9-CM<br>Codes (Description)	ICD-10-CM/PCS<br>Codes (Description)
		O89.0x (Pulmonary complications of anesthesia during the puerperium)
		O89.1 (Cardiac complications of anesthesia during the puerperium)
		O89.2 (Central nervous system complications of anesthesia during the puerperium)
		T88.2XXA (Shock due to anesthesia, initial encounter)
		T88.3XXA (Malignant hyperthermia due to anesthesia, initial encounter)
**14. Sepsis (DX)**	038.xx (Septicemia, specified and unspecified)	A32.7 (Listerial sepsis)
	449 (Septic arterial embolism)	A40.x (Streptococcal sepsis)
	785.52 (Septic shock)	A41.x (Other sepsis)
	995.91 (Sepsis)	I76 (Septic arterial embolism)
	995.92 (Severe sepsis)	O85 (Puerperal sepsis)
	998.02 (Postoperative shock, septic)	O86.04 (Sepsis following an obstetrical procedure)
	670.2x (Puerperal sepsis; used after 10/1/2009)	R65.20 (Severe sepsis without septic shock)
		R65.21 (Severe sepsis with septic shock)
		T81.12XA (Postprocedural septic shock, initial encounter)
		T81.44XA (Sepsis following a procedure)
**15. Shock (DX)**	669.1x (Shock during or following labor and delivery)	O75.1 (Shock during or following labor and delivery)
	785.50 (Shock, unspecified)	R57.x (Shock, not elsewhere classified)
	785.51 (Cardiogenic shock)	T78.2XXA (Anaphylactic shock, unspecified, initial encounter)
	785.59 (Other shock without mention of trauma)	T81.10XA (Postprocedural shock unspecified, initial encounter)
	995 (Other anaphylactic reaction)	T81.11XA (Postprocedural cardiogenic shock, initial encounter)
	998.00 (Postoperative shock, unspecified)	T81.19XA (Other postprocedural shock, initial encounter)
	998.01 (Postoperative shock, cardiogenic)	T88.6XXA (Anaphylactic reaction due to adverse effect of correct drug or medicament properly administered, initial encounter)
	998.09 (Postoperative shock, other)	
**16. Sickle cell disease with crisis (DX)**	282.42 (Sickle-cell thalassemia with crisis)	D57.0x (Hb-SS disease with crisis)
	282.62 (Hb-SS disease with crisis)	D57.21x (Sickle cell/Hb-C disease with crisis)
	282.64 (Sickle-cell/Hb-C disease with crisis)	D57.41x (Sickle cell thalassemia with crisis)
	282.69 (Other sickle-cell disease with crisis)	D57.81x (Other sickle cell disorders with crisis)
	289.52 (Splenic sequestration)	
**17. Air and thrombotic embolism (DX)**	415.0 (Acute cor pulmonale)	I26.x (Pulmonary embolism)
	415.1x (Pulmonary embolism)	O88.012-O88.03 (Obstetric air embolism)

© 2022 Hirai AH et al. *JAMA Network Open.*

Severe Maternal Morbidity Indicator	ICD-9-CM<br>Codes (Description)	ICD-10-CM/PCS<br>Codes (Description)
	673.0x (Obstetrical air embolism)	O88.212-O88.23 (Obstetric thromboembolism)
	673.2x (Obstetrical blood-clot embolism)	O88.312-O88.33 (Obstetric pyemic and septic embolism)
	673.3x (Obstetrical pyemic and septic embolism)	O88.812-O88.83 (Other obstetric embolism)
	673.8x (Other obstetrical pulmonary embolism)	T80.0XXA (Air embolism following infusion, transfusion and therapeutic injection, initial encounter)
**19. Hysterectomy (PR)**	68.39 (Other and unspecified subtotal abdominal hysterectomy)	0UT90ZZ (Resection of Uterus, Open Approach)
	68.49 (Other and unspecified total abdominal hysterectomy)	0UT90ZL (Resection of Uterus, Supracervical, Open Approach)
	68.59 (Other and unspecified vaginal hysterectomy)	0UT97ZL (Resection of Uterus, Supracervical, Via Natural or Artificial Opening)
	68.69 (Other and unspecified radical abdominal hysterectomy)	0UT97ZZ (Resection of Uterus, Via Natural or Artificial Opening)
	68.79 (Other and unspecified radical vaginal hysterectomy)	
	68.9 (Other and unspecified hysterectomy)	
**20. Temporary tracheostomy (PR)**	31.1 (Temporary tracheostomy)	0B110F4 (Bypass trachea to cutaneous with tracheostomy device, open approach)
		0B113F4 (Bypass trachea to cutaneous with tracheostomy device, percutaneous approach)
		0B114F4 (Bypass trachea to cutaneous with tracheostomy device, percutaneous endoscopic approach)
**21.Ventilation (PR)**	96.70 (Continuous invasive mechanical ventilation of unspecified duration)	5A1935Z (Respiratory ventilation, less than 24 consecutive hours)
	96.71 (Continuous invasive mechanical ventilation for less than 96 consecutive hours)	5A1945Z (Respiratory ventilation, 24-96 consecutive hours)
	96.72 (Continuous invasive mechanical ventilation for 96 consecutive hours or more)	5A1955Z (Respiratory ventilation, greater than 96 consecutive hours)

DX: Diagnosis

PR: Procedure

© 2022 Hirai AH et al. *JAMA Network Open.*

eTable 2. Severe Maternal Morbidity per 10 000 Delivery Hospitalizations by State, 2012-2019

State	2012	2013	2014	2015[a] Q1-Q3	2016	2017	2018	2019	Run Line	Total Change 2019 v. 2012[b]		Change with ICD-10-CM/PCS[c]	
										RD	(95% CI)	RD	(95% CI)
AK	70.1	NA	NA	88.4	93.1	106.2	91.3	99.7		29.6	(0.4 , 58.8)	-6.8	(-39.8 , 26.2)
AR	49.5	57.6	56.8	58.8	63.4	57.1	69.8	58.6		9.1	(-1.6 , 19.9)	-0.5	(-11.9 , 11.0)
AZ	64.6	66.7	64.2	66.5	74.7	67.4	67.5	77.8		13.2	(4.8 , 21.5)	4.0	(-4.2 , 12.2)
CA	67.2	70.0	71.0	71.9	75.1	86.4	94.3	99.4		32.2	(28.4 , 36.0)	-1.3	(-4.8 , 2.3)
CO	87.0	86.4	80.4	81.2	74.9	77.7	80.0	90.7		3.7	(-7.1 , 14.4)	-9.0	(-19.0 , 1.0)
CT	56.3	65.6	62.0	75.1	74.6	75.9	87.6	91.8		35.5	(22.6 , 48.5)	-6.2	(-18.9 , 6.5)
DC	NA	94.1	70.5	84.7	88.4	97.3	98.1	98.3				5.6	(-24.3 , 35.5)
FL	68.3	70.3	72.2	77.7	72.4	73.0	73.4	81.8		13.5	(8.2 , 18.8)	-4.3	(-9.6 , 0.9)
GA	81.0	87.2	80.2	69.4	80.1	75.9	80.7	87.2		6.2	(-1.1 , 13.5)	2.9	(-4.1 , 10.0)
HI	60.8	54.8	76.8	66.8	87.9	84.7	104.3	104.8		44.0	(23.0 , 65.0)	11.5	(-8.7 , 31.6)
IA	48.9	56.4	45.9	54.4	53.4	56.3	54.3	56.0		7.1	(-3.4 , 17.5)	4.2	(-6.5 , 14.8)
IL	73.1	74.4	78.4	89.3	81.0	77.5	85.2	89.5		16.4	(9.7 , 23.0)	-7.8	(-14.3 , -1.3)
IN	59.0	64.6	70.8	69.2	72.0	68.5	68.5	67.5		8.5	(0.6 , 16.4)	-3.2	(-11.6 , 5.1)
KS	59.8	61.9	60.7	70.6	56.1	57.7	62.1	66.2		6.4	(-5.5 , 18.2)	-10.5	(-22.4 , 1.3)
KY	69.7	75.7	67.0	71.0	65.2	65.8	75.2	70.0		0.3	(-10.0 , 10.6)	-1.0	(-11.3 , 9.3)
LA	70.1	78.6	82.5	89.5	80.8	76.8	71.7	74.9		4.8	(-5.1 , 14.8)	-11.9	(-22.3 , -1.5)
MA	60.6	63.7	68.3	74.3	88.4	89.5	105.2	100.1		39.5	(29.9 , 49.1)	12.6	(2.9 , 22.3)
MD	81.2	77.7	73.2	67.7	66.0	80.3	82.9	79.3		-1.9	(-11.7 , 7.9)	1.4	(-7.7 , 10.4)
ME	53.8	53.5	79.8	55.3	52.9	59.5	56.6	68.0		14.2	(-5.9 , 34.3)	-19.1	(-39.6 , 1.5)
MI	77.6	73.4	72.6	69.4	76.6	71.2	76.2	78.3		0.7	(-6.8 , 8.2)	5.5	(-1.8 , 12.8)
MN	56.4	59.8	63.7	65.3	61.6	71.1	84.1	87.5		31.1	(21.6 , 40.5)	-8.1	(-16.9 , 0.7)
MO	74.5	67.6	65.9	61.2	70.2	75.4	80.1	83.2		8.7	(-0.6 , 18.0)	11.4	(2.7 , 20.0)
MS	NA	72.7	80.8	78.8	63.1	80.6	74.7	72.4				-12.9	(-27.7 , 1.9)
MT	71.6	66.1	50.4	64.8	57.5	37.4	38.6	52.8		-18.8	(-40.5 , 2.9)	-9.2	(-29.1 , 10.6)
NC	75.7	67.0	69.3	69.3	81.7	76.0	74.0	76.9		1.2	(-6.1 , 8.6)	12.4	(5.3 , 19.5)
ND	54.5	45.9	65.9	45.9	45.0	61.7	47.2	57.1		2.6	(-18.7 , 23.9)	-5.4	(-25.4 , 14.7)
NE	47.1	45.6	52.9	57.3	50.2	52.4	52.6	65.2		18.1	(4.6 , 31.5)	-11.0	(-23.7 , 1.7)
NJ	67.8	71.4	73.5	71.1	73.2	74.4	81.3	80.7		12.9	(5.3 , 20.5)	-4.2	(-11.9 , 3.4)
NM	92.1	83.7	81.5	81.8	82.5	82.2	74.0	80.0		-12.1	(-29.0 , 4.9)	4.5	(-11.9 , 20.9)
NV	69.3	63.5	85.1	73.1	60.6	69.1	65.2	84.4		15.1	(1.7 , 28.4)	-21.5	(-34.2 , -8.7)
NY	86.3	88.3	94.9	93.2	80.0	83.5	88.5	92.2		5.9	(0.3 , 11.5)	-17.0	(-22.6 , -11.5)
OH	79.0	85.1	84.4	83.3	76.8	72.6	77.8	78.4		-0.6	(-7.4 , 6.2)	-10.8	(-17.7 , -3.8)
OK	60.0	59.0	64.1	63.9	65.9	76.5	73.0	68.8		8.8	(-1.5 , 19.1)	5.5	(-5.0 , 15.9)
OR	56.9	65.4	63.7	67.3	61.6	66.7	67.0	72.2		15.3	(4.1 , 26.5)	-8.3	(-19.4 , 2.7)
PA	70.4	66.9	71.7	77.4	74.2	76.4	77.0	87.8		17.4	(10.5 , 24.3)	-5.4	(-11.9 , 1.2)
RI	76.3	68.2	58.6	47.0	80.0	100.4	85.3	111.3		35.0	(8.4 , 61.6)	41.6	(18.7 , 64.5)
SC	76.7	83.7	78.2	81.1	69.8	72.7	79.6	72.5		-4.2	(-14.4 , 6.0)	-9.9	(-20.4 , 0.6)
SD	33.1	45.5	47.7	39.4	40.4	47.0	57.1	52.5		19.4	(2.0 , 36.9)	-4.4	(-22.4 , 13.6)
TN	96.1	106.2	90.9	90.8	73.8	79.7	79.4	73.1		-23.0	(-32.7 , -13.2)	-14.7	(-25.0 , -4.4)
TX	64.7	69.6	70.8	67.8	66.1	62.9	66.2	72.4		7.7	(3.9 , 11.6)	-7.0	(-10.8 , -3.2)
UT	46.4	46.9	52.6	55.9	53.7	60.2	47.3	53.7		7.3	(-1.8 , 16.4)	-1.3	(-10.6 , 8.1)
VA	70.7	67.0	70.2	68.5	70.6	64.1	69.2	66.9		-3.8	(-11.4 , 3.8)	0.7	(-6.9 , 8.3)
VT	71.8	62.7	72.3	65.6	65.8	62.7	60.4	76.6		4.8	(-28.9 , 38.5)	-1.6	(-34.7 , 31.5)
WA	51.5	55.9	56.8	56.6	58.7	60.6	66.8	68.2		16.7	(9.0 , 24.5)	-1.4	(-8.9 , 6.2)
WI	48.3	45.9	47.1	49.1	61.3	54.9	62.3	65.4		17.1	(8.6 , 25.5)	9.2	(1.2 , 17.2)
WV	71.3	76.6	85.3	79.3	86.0	70.1	85.5	83.7		12.4	(-5.7 , 30.5)	0.9	(-17.8 , 19.6)
WY	63.9	73.4	78.5	44.0	70.0	47.9	52.4	84.5		20.6	(-9.9 , 51.1)	0.1	(-27.4 , 27.7)

© 2022 Hirai AH et al. *JAMA Network Open.*

Source: Agency for Healthcare Research and Quality, Healthcare Cost and Utilization Project, State Inpatient Databases, 2012-2019
ICD-10-CM/PCS: International Classification of Disease, 10th Revision, Clinical Modification and Procedure Coding System
RD: Rate Difference; significant increases highlighted in orange and significant decreases highlighted in blue, $p<0.05$.
NA = Not Available; State did not participate in the Healthcare Cost and Utilization Project
[a] Excludes the fourth quarter to distinguish the transition to ICD-10-CM) in October 2015.
[b] Total change is the absolute rate difference (2019 minus 2012).
[c] Change with ICD-10-CM/PCS is the immediate change associated with transition obtained from segmented linear binomial regression models controlling for quarter and quarterly time trends allowed to vary before and after transition.

© 2022 Hirai AH et al. *JAMA Network Open.*

eTable 3. Disseminated Intravascular Coagulation per 10 000 Delivery Hospitalizations by State, 2012-2019

State	2012	2013	2014	2015[a] Q1-Q3	2016	2017	2018	2019	Run Line	Total Change 2019 v. 2012[b]		Change with ICD-10-CM/PCS[c]	
										RD	(95% CI)	RD	(95% CI)
AK	29.1	NA	NA	27.0	29.5	28.6	19.9	14.1		-15.0	(-29.7 , -0.3)	6.7	(-12.2 , 25.5)
AR	14.3	16.3	17.1	24.7	18.3	12.0	15.3	12.9		-1.4	(-6.7 , 4.0)	-7.9	(-14.0 , -1.7)
AZ	34.9	29.1	26.0	27.2	17.9	14.2	15.0	16.8		-18.1	(-23.1 , -13.1)	-6.8	(-11.5 , -2.1)
CA	30.6	31.1	29.9	28.3	21.6	25.3	24.8	28.7		-1.9	(-4.2 , 0.3)	-6.7	(-8.8 , -4.6)
CO	55.3	42.8	39.3	39.7	25.0	22.8	22.7	24.9		-30.4	(-37.6 , -23.2)	-11.5	(-17.9 , -5.1)
CT	17.7	23.2	20.6	16.8	17.7	21.4	24.5	27.2		9.5	(2.3 , 16.6)	-1.4	(-8.2 , 5.5)
DC	NA	48.7	26.0	33.6	25.0	27.8	29.0	31.6				-4.5	(-22.0 , 13.0)
FL	28.0	29.6	27.3	29.0	19.9	20.0	19.6	22.3		-5.7	(-8.8 , -2.6)	-9.0	(-12.0 , -5.9)
GA	39.2	42.8	35.2	25.1	24.7	22.7	21.2	21.6		-17.6	(-21.9 , -13.2)	-1.8	(-6.1 , 2.5)
HI	23.0	21.3	26.5	24.6	32.0	28.7	47.6	44.5		21.5	(8.1 , 34.9)	1.6	(-10.2 , 13.4)
IA	21.0	24.5	17.3	19.9	15.3	12.6	13.2	15.4		-5.6	(-11.7 , 0.6)	-3.6	(-9.6 , 2.4)
IL	33.2	33.4	33.7	35.9	24.7	26.9	28.7	31.2		-2.0	(-6.1 , 2.2)	-11.0	(-15.1 , -7.0)
IN	25.6	28.0	28.4	27.9	22.3	19.9	21.6	22.7		-2.9	(-7.8 , 2.0)	-9.8	(-14.9 , -4.8)
KS	28.9	30.8	18.3	26.3	13.1	11.8	12.1	13.7		-15.2	(-22.0 , -8.4)	-9.1	(-15.6 , -2.5)
KY	35.6	34.2	29.8	31.5	15.7	20.4	24.3	20.4		-15.2	(-21.7 , -8.8)	-10.8	(-17.1 , -4.5)
LA	33.2	35.6	38.3	34.0	25.3	25.5	22.5	19.5		-13.7	(-19.7 , -7.7)	-10.3	(-16.8 , -3.9)
MA	20.6	22.0	22.2	21.4	26.6	24.4	35.4	29.0		8.4	(3.1 , 13.7)	2.2	(-3.2 , 7.6)
MD	34.4	33.5	25.5	19.4	16.4	21.9	22.0	22.8		-11.6	(-17.4 , -5.8)	-1.5	(-6.5 , 3.5)
ME	19.9	21.2	26.0	15.8	NR	NR	13.1	12.7		-7.2	(-17.4 , 3.1)	-13.8	(-24.5 , -3.2)
MI	31.5	30.3	28.3	21.1	18.9	16.7	17.8	19.1		-12.4	(-16.7 , -8.2)	-3.3	(-7.4 , 0.8)
MN	21.1	24.3	21.2	19.9	17.9	19.1	20.4	18.4		-2.7	(-7.7 , 2.3)	-2.4	(-7.4 , 2.6)
MO	33.3	27.9	23.3	22.3	16.8	18.9	21.9	21.9		-11.4	(-16.9 , -5.9)	-1.9	(-6.8 , 2.9)
MS	NA	26.0	26.8	23.3	15.1	25.2	17.2	18.1				-4.0	(-12.1 , 4.2)
MT	24.5	30.7	20.9	25.2	11.9	13.1	NR	11.7		-12.8	(-24.5 , -1.1)	-12.9	(-25.1 , -0.7)
NC	36.3	33.5	26.2	25.5	28.4	20.8	21.8	21.5		-14.8	(-19.4 , -10.3)	4.8	(0.6 , 9.1)
ND	24.5	25.1	26.7	19.9	20.2	30.8	12.8	16.0		-8.5	(-21.4 , 4.4)	2.7	(-10.6 , 16.0)
NE	17.3	17.2	24.5	13.5	13.0	13.3	9.5	14.8		-2.5	(-9.7 , 4.6)	-5.6	(-12.8 , 1.6)
NJ	26.3	27.7	26.0	26.8	22.8	22.6	22.3	22.4		-3.9	(-8.3 , 0.4)	-3.1	(-7.6 , 1.4)
NM	50.6	44.1	35.9	39.3	26.2	23.2	20.7	18.6		-32.0	(-42.6 , -21.5)	-6.7	(-17.0 , 3.6)
NV	35.2	33.2	36.8	32.9	12.6	14.3	18.7	20.3		-14.9	(-22.9 , -6.9)	-21.3	(-29.0 , -13.6)
NY	40.9	42.5	45.5	40.0	25.5	28.2	27.8	30.0		-10.9	(-14.4 , -7.4)	-16.7	(-20.2 , -13.1)
OH	40.5	43.9	41.2	35.1	22.2	22.0	20.6	20.9		-19.6	(-23.8 , -15.4)	-13.8	(-18.2 , -9.4)
OK	19.5	18.0	21.0	21.9	16.1	15.3	16.9	13.6		-5.9	(-11.0 , -0.7)	-6.0	(-11.6 , -0.4)
OR	25.8	26.8	25.8	27.2	17.5	19.7	24.8	22.3		-3.5	(-10.2 , 3.3)	-10.1	(-16.6 , -3.5)
PA	28.3	25.0	23.9	29.3	20.6	19.8	20.5	23.6		-4.7	(-8.6 , -0.8)	-6.0	(-9.7 , -2.3)
RI	18.6	22.7	22.7	16.1	31.1	44.0	31.0	47.2		28.6	(12.8 , 44.4)	16.5	(1.9 , 31.1)
SC	30.5	37.9	32.2	32.5	18.4	20.3	19.0	15.9		-14.6	(-20.3 , -8.9)	-14.8	(-21.1 , -8.4)
SD	19.3	12.7	19.8	NR	11.4	NR	13.4	12.0		-7.3	(-17.8 , 3.2)	-1.0	(-10.2 , 8.3)
TN	56.1	61.2	46.2	44.1	24.8	27.0	22.9	20.5		-35.6	(-42.2 , -29.0)	-16.2	(-23.0 , -9.3)
TX	29.5	28.7	29.7	26.5	18.5	17.3	17.1	16.9		-12.6	(-14.8 , -10.3)	-8.5	(-10.8 , -6.2)
UT	19.8	21.5	18.3	23.4	15.9	16.6	12.4	11.6		-8.2	(-13.3 , -3.1)	-3.9	(-9.4 , 1.7)
VA	35.5	30.4	29.9	27.3	19.7	17.0	18.8	18.5		-17.0	(-21.8 , -12.2)	-7.3	(-11.8 , -2.8)
VT	27.9	NR	23.5	NR	37.0	23.0	NR	NR				23.5	(5.1 , 41.9)
WA	19.6	22.9	22.4	20.6	17.7	17.8	18.0	17.7		-1.9	(-6.2 , 2.4)	-5.0	(-9.5 , -0.4)
WI	20.0	15.7	14.7	11.6	18.6	16.3	15.5	16.9		-3.1	(-7.9 , 1.7)	7.7	(3.4 , 12.0)
WV	29.6	33.5	37.0	34.8	20.6	17.0	20.3	16.9		-12.7	(-22.5 , -2.9)	-17.5	(-28.7 , -6.3)
WY	30.6	33.2	30.8	NR	NR	NR	NR	21.6		-9.0	(-26.7 , 8.7)	-7.0	(-22.4 , 8.5)

© 2022 Hirai AH et al. *JAMA Network Open.*

Source: Agency for Healthcare Research and Quality, Healthcare Cost and Utilization Project, State Inpatient Databases, 2012-2019
ICD-10-CM/PCS: International Classification of Disease, 10th Revision, Clinical Modification and Procedure Coding System
RD: Rate Difference; significant increases highlighted in orange and significant decreases highlighted in blue, $p<0.05$.
NA = Not Available; State did not participate in the Healthcare Cost and Utilization Project
NR = Not Reportable; Suppressed to protect confidentiality, ≤10 cases
[a] Excludes the fourth quarter to distinguish the transition to ICD-10-CM) in October 2015.
[b] Total change is the absolute rate difference (2019 minus 2012).
[c] Change with ICD-10-CM/PCS is the immediate change associated with transition obtained from segmented linear binomial regression models controlling for quarter and quarterly time trends allowed to vary before and after transition.

© 2022 Hirai AH et al. *JAMA Network Open.*
